# Vanadium Pentoxide Nanofibers/Carbon Nanotubes Hybrid Film for High-Performance Aqueous Zinc-Ion Batteries

**DOI:** 10.3390/nano11041054

**Published:** 2021-04-20

**Authors:** Xianyu Liu, Liwen Ma, Yehong Du, Qiongqiong Lu, Aikai Yang, Xinyu Wang

**Affiliations:** 1 School of Chemistry and Chemical Engineering, Lanzhou City University, Lanzhou 730070, China; xyliu15@mail.ustc.edu.cn; 2 Institute of Materials and Technology, Dalian Maritime University, Dalian 116026, China; maliwen2019@163.com (L.M.); duyhdlmu@foxmail.com (Y.D.); 3 Leibniz Institute for Solid State and Materials Research (IFW) Dresden e.V., Helmholtzstraße 20, 01069 Dresden, Germany; 4 Forschungszentrum Jülich GmbH, Institute of Energy and Climate Research, Materials Synthesis and Processing (IEK-1), 52425 Jülich, Germany; a.yang@fz-juelich.de

**Keywords:** aqueous zinc-ion battery, vanadium pentoxide, carbon nanotubes, hybrid film

## Abstract

Aqueous zinc-ion batteries (ZIBs) with the characteristics of low production costs and good safety have been regarded as ideal candidates for large-scale energy storage applications. However, the nonconductive and non-redox active polymer used as the binder in the traditional preparation of electrodes hinders the exposure of active sites and limits the diffusion of ions, compromising the energy density of the electrode in ZIBs. Herein, we fabricated vanadium pentoxide nanofibers/carbon nanotubes (V_2_O_5_/CNTs) hybrid films as binder-free cathodes for ZIBs. High ionic conductivity and electronic conductivity were enabled in the V_2_O_5_/CNTs film due to the porous structure of the film and the introduction of carbon nanotubes with high electronic conductivity. As a result, the batteries based on the V_2_O_5_/CNTs film exhibited a higher capacity of 390 mAh g^−1^ at 1 A g^−1^, as compared to batteries based on V_2_O_5_ (263 mAh g^−1^). Even at 5 A g^−1^, the battery based on the V_2_O_5_/CNTs film maintained a capacity of 250 mAh g^−1^ after 2000 cycles with a capacity retention of 94%. In addition, the V_2_O_5_/CNTs film electrode also showed a high energy/power density (e.g., 67 kW kg^−1^/267 Wh kg^−1^). The capacitance response and rapid diffusion coefficient of Zn^2+^ (~10^−8^ cm^−2^ s^−1^) can explain the excellent rate capability of V_2_O_5_/CNTs. The vanadium pentoxide nanofibers/carbon nanotubes hybrid film as binder-free cathodes showed a high capability and a stable cyclability, demonstrating that it is highly promising for large-scale energy storage applications.

## 1. Introduction

The lithium-ion battery is widely used in daily life owing to its many advantages including a high operating voltage, high specific capacity, and long cycle life [1,2]. However, lithium resources on the earth are limited, and the contradiction between its high price and increasing demand is becoming increasingly prominent. In addition, lithium-ion batteries suffer other issues such as high internal resistance, harmful organic electrolytes, and safety hazards [3,4]. These problems restrict their large-scale applications. Rechargeable aqueous batteries have the merits of low production costs, and the electrolyte used is an aqueous electrolyte with high safety. Therefore, it is expected to supplement lithium-ion batteries for new-generation electrochemical energy storage systems [5,6,7,8].

Among rechargeable aqueous batteries, aqueous zinc-ion batteries (ZIBs) have attracted more attention due to the high abundance of metal zinc in the earth’s resources, low cost, and nontoxicity [9,10]. As zinc metal foil can be directly used as the anode, the development of cathodes of ZIBs have become a research hotspot. The reported cathode materials for ZIBs mainly include manganese compounds, vanadium oxides, Prussian blue, and organic compounds [11,12,13,14]. Among these cathode materials, vanadium pentoxide (V_2_O_5_) has a unique layered structure with a wide range of valence states (from V^3+^ to V^5+^), which is conducive to the multielectron transfer providing a high specific capacity [15,16]. However, its ion conductivity is low and its diffusion kinetics is slow, resulting in a poor rate performance and unsatisfied cycle performance. Furthermore, the nonconductive and non-redox active polymer was used as the binder in the traditional preparation of electrodes, which hinders the diffusion of zinc ions and compromises the energy density of the electrode [17]. Therefore, in order to avoid using binders, it is important to design a binder-free V_2_O_5_ electrode.

In this work, V_2_O_5_ nanofibers/carbon nanotubes (V_2_O_5_/CNTs) hybrid films were fabricated and employed as the cathode of ZIBs, and the usage of nonconductive and non-redox active binders was avoided. The network structure of V_2_O_5_/CNTs film is helpful for improving the electronic and ionic conductivity of the electrode. Compared with batteries with binders, the batteries based on the V_2_O_5_/CNTs film showed a higher specific capacity and a better cycle stability. This work proved that the electrochemical performance of ZIBs can be improved by the application of binder-free electrodes.

## 2. Materials and Methods

### 2.1. Preparation of V_2_O_5_ Nanofibers

First, 0.75 g of NH_4_VO_3_ (99%, Aladdin) and 1.25 g of P123 (Sigma-Aldrich, St. Louis, MO, USA) were dissolved in 75 mL of water containing 3.75 mL of 2 M of HCl. The mixture was stirred at room temperature for 7 h and then transferred into a Teflon autoclave. After the autoclave was sealed, it was held at 120 °C for 24 h and then cooled to room temperature. The product was washed with deionized water several times and then freeze-dried to obtain V_2_O_5_ nanofibers.

### 2.2. Preparation of V_2_O_5_/CNTs Hybrid Film Electrodes

Here, 20 mg of V_2_O_5_ and 15 mg of CNTs (length: 0.5–1.5 μm; diameter ∼5 nm; Carbon Solutions Inc., Riverside, CA, USA) were dissolved in 40 mL of DMF; then, the mixture was sonicated to form a mixed suspension. The V_2_O_5_/CNTs film was fabricated by filtration and then dried in an oven at 80 °C.

### 2.3. Material Characterizations

Scanning electron microscopy (SEM, Supra-55, Zeiss, Oberkochen, Germany) and transmission electron microscopy (TEM, JEOL2100F, JEOL, Tokyo, Japan) were used to investigate the morphology of the samples. The chemical components of the V2O5/CNTs film were confirmed with X-ray photoelectron spectroscopy (XPS, PHI 1600 ESCA, PerkinElmer, Waltham, MA, USA). The structure of the V_2_O_5_ nanowires and V_2_O_5_/CNTs film was characterized using X-ray diffraction (XRD, Rigaku D/Max-3A, Rigaku Corporation, Tokyo, Japan). Raman spectra were recorded by a spectrophotometer (Thermo-Fisher Scientific, Waltham, MA, USA).

### 2.4. Electrochemical Measurements

Stainless-steel CR2032 coin cells were assembled and tested to evaluate the electrochemical performance of the samples. The cells were assembled using a V_2_O_5_/CNTs composite film as the cathode, filter paper as the separator, Zn foil as the anode, and 3 M of aqueous Zn(CF_3_SO_3_)_2_ solution as the electrolyte. Electrochemical impedance spectroscopy (EIS) was performed using a frequency range between 10 mHz and 100 kHz with an AC perturbation signal of 10 mV. Cyclic voltammetry (CV) of the as-assembled battery was conducted at various scan rates (0.2–1.0 mV·s^−1^). A CHI 660E electrochemical workstation (Shanghai Chenhua, Shanghai, China) was employed to record the CV and EIS results. A CT2001A LAND electrochemical workstation was used to perform the galvanostatic intermittent titration technique (GITT), galvanostatic charge/discharge (GCD), and cyclic performance, within a voltage window of 0.3–1.5 V. All specific capacities reported in this work are based on the cathode mass.

## 3. Results

**Figure 1:**
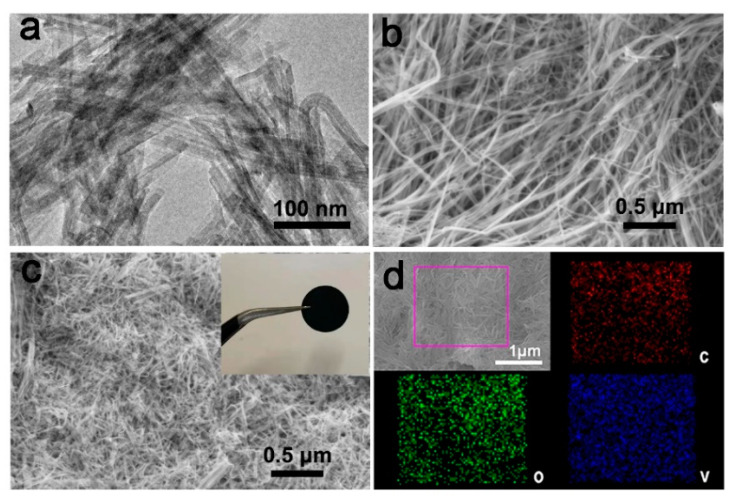
(**a**) TEM image of the V_2_O_5_ nanofibers. (**b**) SEM image of V_2_O_5_ nanofibers. (**c**) SEM image and optical image (inset) of V_2_O_5_/CNTs films. (**d**) Element mappings of V_2_O_5_/CNTs.

**Figure 2:**
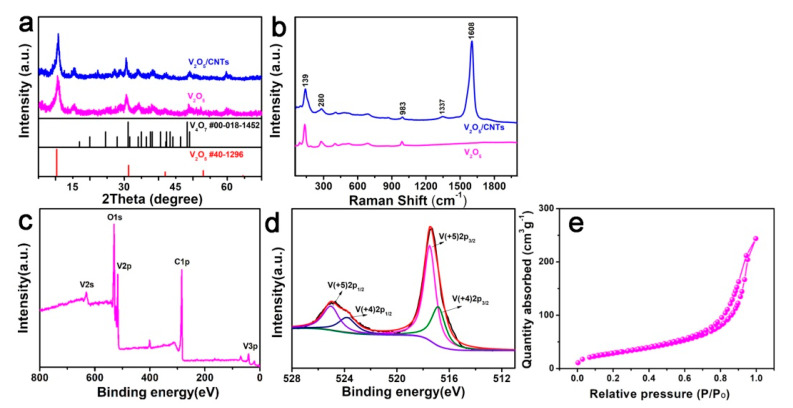
(**a**) XRD patterns of V_2_O_5_ and V_2_O_5_/CNTs. (**b**) Raman spectra of the V_2_O_5_ and V_2_O_5_/CNTs. (**c**) XPS spectra of V_2_O_5_/CNTs and (**d**) V 2p spectrum. (**e**) Nitrogen adsorption/desorption isotherms.

The morphology of the as-prepared V_2_O_5_ was investigated with a transmission electron microscope (TEM) and scanning electron microscope (SEM). The TEM and SEM images reveal that the V_2_O_5_ had a nanofiber morphology with a diameter of ~18 nm and lengths of several micrometers (Figure 1a,b). After being mixed with CNTs, the V_2_O_5_ nanofibers were embedded into the network of CNTs (Figure 1c). Furthermore, the V_2_O_5_/CNTs electrode showed a freestanding structure (inset of Figure 1c). Elemental mappings confirmed that C, O, and V elements were evenly distributed in the V_2_O_5_/CNTs nanobelts (Figure 1d). XRD and Raman spectroscopy tests were further performed to investigate the V_2_O5 nanofibers and V_2_O_5_/CNTs film. XRD patterns of the V_2_O_5_ nanofibers and V_2_O_5_/CNTs film presented typical (001) and (003) peaks (Figure 2a), which fitted well with the layered V_2_O_5_ (JCPDS no. 40–1296). Peaks of V_4_O_7_ were also detected, which may be ascribed to the reduction of V_2_O_5_ by P123 [18]. The Raman spectrum of V_2_O_5_/CNTs showed the presence of D and G peaks as compared to that of V_2_O_5_, indicating the presence of CNTs in composite films [19]. The three peaks located at 139, 280, and 983 cm^−1^ are assigned to the V-O vibration in both the V_2_O_5_/CNTs and V_2_O_5_ samples (Figure 2b) [20]. In addition, in the XPS survey spectrum, solely C, V, and O elements were detected, confirming the purity of the as-prepared V_2_O_5_/CNTs sample (Figure 2c). The peak located at 517.5 eV in the V 2p_1/2_ spectrum and the peak at 525.2 eV in the V 2p_3/2_ spectrum correspond to V^5+^, and the peak located at 516.8 eV in the V 2p_1/2_ spectrum and the peak at 523.7 eV is related to V^4+^ (Figure 2d) [18]. The surface area of V_2_O_5_/CNTs hybrid films was measured to be 107 m^2^ g^−1^, as shown in Figure 2e.

**Figure 3:**
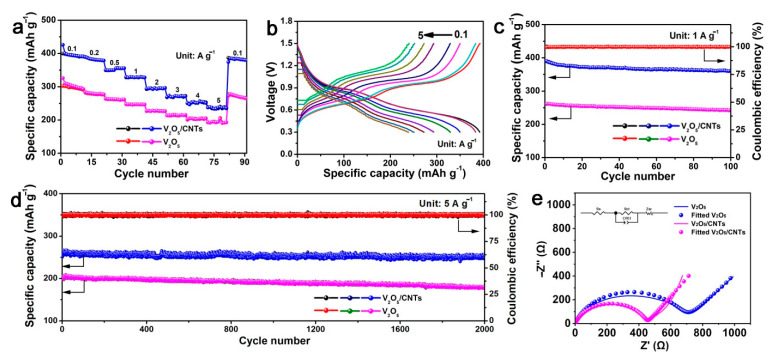
(**a**) The rate performance of the V_2_O_5_/CNTs film and V_2_O_5_ electrodes. (**b**) Charge/discharge curves of the V_2_O_5_/CNTs film and V_2_O_5_ electrodes at different current densities. (**c**) Cycle performance of V_2_O_5_/CNTs film and V_2_O_5_ electrodes. (**d**) Long-term cycling performance of V_2_O_5_/CNTs film and V_2_O_5_ electrodes at 5 A g^−1^. (**e**) Nyquist plots of V_2_O_5_/CNTs film and V_2_O_5_ electrodes.

The electrochemical properties of V_2_O_5_ and V_2_O_5_/CNTs films were further evaluated in ZIBs. The specific capacity at different current densities of V_2_O_5_ and V_2_O_5_/CNTs samples are shown in Figure 3a. The V_2_O_5_/CNTs film showed a high capacity of 399 mAh g^−1^ at 0.1 A g^−1^, which is higher than that of the V_2_O_5_ nanofiber (312 mAh g^−1^). The reason for the capacity decreasing at low current densities is ascribed to the continuous V_2_O_5_ dissolution [5]. Even at a high current density of 5 A g^−1^, the V_2_O_5_/CNTs film still exhibited a high discharge capacity of 239 mAh g^−1^, while the V_2_O_5_ nanofiber showed a capacity of 187 mAh g^−1^. The result demonstrates that the V_2_O_5_/CNTs film showed a higher rate capability than that of V_2_O_5_ nanofibers electrode due to the introduction of CNTs. Figure 3b displays the charge/discharge curves of the V_2_O_5_/CNTs film under various current densities. The charge/discharge curves at different current densities showed similar shapes, indicating the fast charge transfer kinetics of the V_2_O_5_/CNTs film.

**Figure 4:**
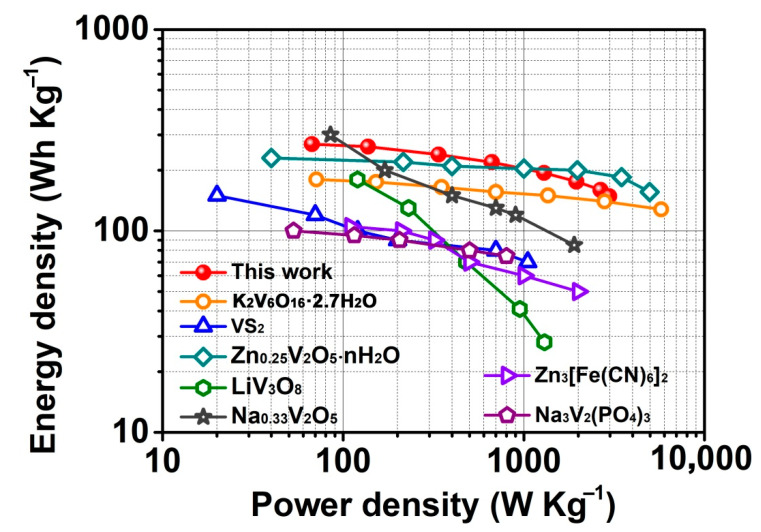
Ragone plot of the batteries based on V_2_O_5_/CNTs compared with oher reported data for ZIBs.

In addition, V_2_O_5_/CNTs films maintain a high discharge capacity of 273 mAh g^−1^ after 100 cycles at 1 A g^−1^ (Figure 3c). Apart from the good rate capability, the V_2_O_5_/CNTs film also displayed an excellent long-term cyclic stability. Even at 5 A g^−1^ over 2000 cycles, the batteries based on the V_2_O_5_/CNTs film maintained a capacity of 251 mAh g^−1^ with a high-capacity retention of 94% (Figure 3d), which is much higher than those of pristine V_2_O_5_ (168 mAh g^−1^ and 81%). The long cycle capability of the V_2_O_5_/CNTs film was comparable or higher than most of the previously reported V-based materials without CNTs (Table 1) [21,22,23,24,25,26,27,28,29,30,31]. Furthermore, compared with other works using CNTs in an V_2_O_5_ electrode, the batteries based on the V_2_O_5_/CNTs film still displayed a comparable capacity and cycle performance (Table 2) [32,33,34]. These superior electrochemical performances could be ascribed to the nanowire V_2_O_5_ knitted with CNTs being helpful for the electrode to keep the close contact and provide an effective electron transmission. The electrochemical impedance spectra (EIS) measurements were performed to study the kinetics. As shown in Figure 3e, both the Nyquist plots of the V_2_O_5_ and V_2_O_5_/CNTs film consisted of a hemicycle at the high-frequency region (charge transfer-limited process) and a straight line in the low-frequency region (ion diffusion-limited process). As for the V_2_O_5_/CNTs sample, the line in the low-frequency region was substantially steeper and the inner diameter of the hemicycle in the high-frequency region was small compared with V_2_O_5_, manifesting that it had a fast ion diffusion rate and a small resistance. The charge transfer resistance (Rct) of the V_2_O_5_/CNTs film electrode was about 462 Ω after fitting, which is smaller than that of V_2_O_5_ (741 Ω), revealing that the introduction of CNTs is beneficial for the high electronic conductivity and efficient Zn^2+^ transport in the V_2_O_5_/CNTs cathode. Furthermore, the energy/power densities were calculated and compared with other cathode materials (Figure 4). Impressively, the batteries based on the V_2_O_5_/CNTs film display a remarkable energy density and an impressive power density (e.g., 267 Wh kg^−1^ and 3.2 kW kg^−1^), which is comparable with the cathodes of K_2_V_6_O_16_·2.7H_2_O, VS_2_, Zn_0.25_V_2_O_5_·nH_2_O, LiV_3_O_8_, Na_0.33_V_2_O_5_, Zn_3_[Fe(CN)_6_]_2_, and Na_3_V_2_(PO_4_)_3_ [27,30,35,36,37,38,39].

**Figure 5:**
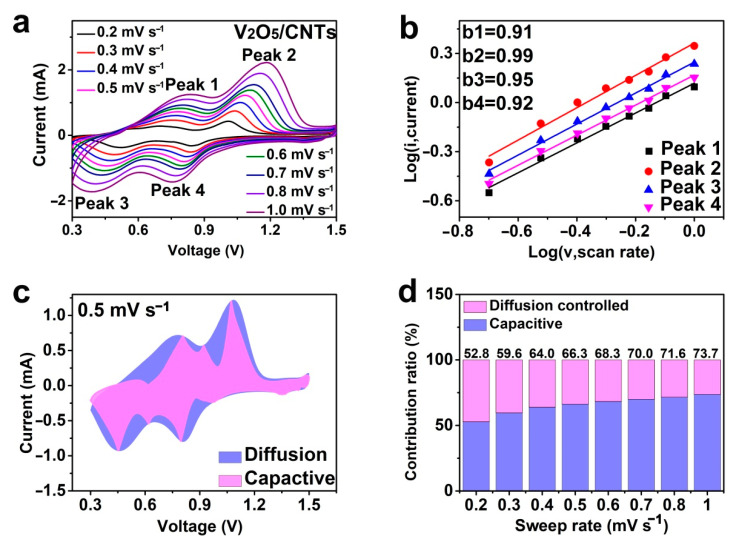
(**a**) CV curves of the V_2_O_5_/CNTs electrode at different scan rates. (**b**) Log(current) vs. log (scan rate) plots of four peaks in the CV curves. (**c**) Capacity separation curves at 0.5 mV·s^−1^. (**d**) Capacity contribution ratios at multiple scan rates.

The electrochemical kinetics was further investigated to explain the electrochemical performance. The CV curves of the V_2_O_5_/CNTs film was measured at different scan rates. As shown in Figure 5a, the CV curves showed similar shapes with the growth of the scan rates, which indicates its good electrochemical reversibility. The characteristic peaks appeared at 0.5/0.7 V, as well as 0.8/1.0 V, reflecting the redox reaction in V_2_O_5_/CNTs (Figure 5a) [15,18]. According to the previous literature, the peak current (i) and scan rates (v) have a linear relationship, which can be written as [40]:

(1) $$i=av^{b}$$

where *a* and *b* are adjustable parameters. When *b* is close to 1, the reaction is a mainly surface-controlled process; when *b* is near to 0.5, the reaction is dominated by diffusion-controlled behavior. The slope of the peaks of the V_2_O_5_/CNTs film is close to 1, which is higher than that of the V_2_O_5_ electrode [15,18,22], indicating that the electrochemical process of the V_2_O_5_/CNTs is dominated by the pseudocapacitive behavior (Figure 5b). Furthermore, the contribution of pseudocapacitance at different scan rates can be calculated by the following equation: [41]

(2) $$i=k_{1}v+k_{2}v^{1/2}$$

The current density (*i*) should be divided into two parts, the pseudocapacity influence (*k*_1_*v*) and the diffusion-dominant reaction (*k*_2_*v*^1/2^). Based on the integration of the CV curve, 66.3% of the total charge storage of the V_2_O_5_/CNTs cathode is from the capacitive contribution at 0.5 mV s^−1^ (Figure 5c). The proportions of the capacitive contribution for the V_2_O_5_/CNTs cathode are listed in Table 3 (Figure 5d).

**Figure 6:**
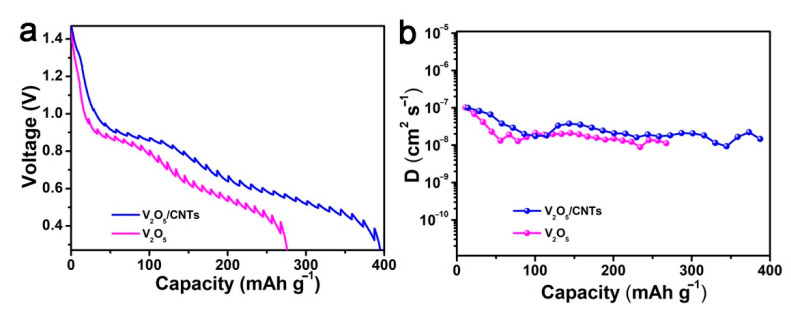
(**a**) GITT measurements and (**b**) the corresponding Zn^2+^ diffusion coefficients of V_2_O_5_/CNTs and V_2_O_5_ in the discharge process.

In order to study the kinetics of Zn^2+^ diffusion in these batteries, a constant-current intermittent titration technique (GITT) test was performed (Figure 6a). The diffusion coefficients (*D*) of Zn^2+^ ions at the discharge process and charge process can be estimated according to the following equation [42]:

(3) $$D=\frac{4}{\pi \tau }{\left(\frac{m_{B}V_{M}}{M_{B}S}\right)}^{2}{\left(\frac{∆E_{s}}{∆E_{\tau }}\right)}^{2}\;\;\left(\tau \ll L^{2}/D\right)$$

where τ is the time for an applied galvanostatic current; *m_B_*, *M_B_*, and *V_M_* are the mass, molecular weight, and molar volume, respectively; *S* is the active surface of the electrode (taken as the geometric area of the electrode); Δ*Es* and Δ*Eτ* are the quasi-equilibrium potential and the change in cell voltage *E* during the current pulse, respectively; *L* is the average radius of the material particles. In our case, the *D*_Zn_ value of the battery with the V_2_O_5_/CNTs film electrode is ~10^−8^ cm^−2^ s^−1^, which is higher than the value of the V_2_O_5_ cathode (Figure 6b), which is consistent with the CV results. Due to the network structure of the V_2_O_5_/CNTs films, high values of the capacitive contribution and diffusion coefficients of Zn^2+^ are enabled, leading to a high rate capability of V_2_O_5_/CNTs films. All the above results conclusively substantiate that V_2_O_5_/CNTs possesses a bright future for the practical application of ZIBs.

## 4. Conclusions

In summary, V_2_O_5_/CNTs films were fabricated and employed as binder-free cathodes for ZIBs. The V_2_O_5_/CNTs film electrodes without nonconductive and non-redox active binders are beneficial for the exposure of active sites and the transfer of electrons and zinc ions, enhancing the electrochemical performance. As a result, the ZIBs based on V_2_O_5_/CNTs film electrodes possess an excellent rate performance and stable cycle life. This work provides a viable method for fabricating freestanding and binder-free electrodes for energy storage devices and other electronics into highly flexible devices.

## Figures and Tables

**Table 1:** The comparison of long-term cycle performances of the V_2_O_5_/CNTs cathode.

Cathodes	Rate (A g^−1^)	Capacity Retention	Final Capacity (mAh g^−1^)	Reference
V_2_O_5_/CNTs	5	94% (2000 cycles)	251	This work
V_2_O_5_·nH_2_O	6	71.0% (900 cycles)	213	[21]
Cu^2+^-V_2_O_5_	10	88.0% (5000 cycles)	180	[22]
K^+^-V_2_O_5_	8	96.0% (1500 cycles)	172	[23]
Graphene/H_2_V_3_O_8_	6	87.0% (2000 cycles)	240	[24]
V_2_O_5_@PANI	5	93.8% (1000 cycles)	201	[25]
2D V_2_O_5_	20	68.2% (500 cycles)	117	[26]
Zn_0.25_V_2_O_5_·nH_2_O	2.4	80.0% (1000 cycles)	208	[27]
NaV_3_O_8_·1.5H_2_O	4	82.0% (1000 cycles)	120	[28]
Na_2_V_6_O_16_·3H_2_O	14	85% (1000 cycles)	129	[29]
K_2_V_6_O_16_·2.7H_2_O	5	88% (229 cycles)	139	[30]
Na_1.1_V_3_O_7.9_/rGO	1	93% (500 cycles)	85	[31]

**Table 2:** The comparison of the V_2_O_5_/CNTs cathode with other CNT-based V_2_O_5_ electrodes.

Cathodes	Specific Capacity	Capacity Retention	Reference
V_2_O_5_/CNTs	399 mAh g^−1^ (0.1 A g^−1^) 327 mAh g^−1^ (1 A g^−1^)	5A g^−1^: 94% (2000 cycles)	This work
V_2_O_5_/CNTs nanopaper	375 mAh g^−1^ (0.5 A g^−1^)	10A g^−1^: 80.0% (500 cycles)	[32]
V_2_O_5_/CNTs (VCP)	312 mAh g^−1^ (1 A g^−1^)	1 A g^−1^: 81% (2000 cycles)	[33]
V_2_O_5_@CNTs	293 mAh g^−1^ (0.3 A g^−1^)	5 A g^−1^: 72.0% (6000 cycles)	[34]

**Table 3:** The proportions of the capacitive contribution for the V_2_O_5_/CNTs cathode.

Scan rate (mV s^−1^)	0.2	0.3	0.4	0.5	0.6	0.7	0.8	1.0
Capacitive contribution (%)	52.8	59.6	64.0	66.3	68.3	70.0	71.6	73.7

## Data Availability

Data is contained within the article.
